# Prevalence and comorbidity of common mental disorders and associations with suicidal ideation in the adult population

**DOI:** 10.4178/epih.e2017031

**Published:** 2017-07-22

**Authors:** Yousef Veisani, Fathola Mohamadian, Ali Delpisheh

**Affiliations:** 1 Psychosocial Injuries Research Center, Ilam University of Medical Sciences, Ilam, Iran; 2 Department of Psychology, Psychosocial Injuries Research Center, Ilam University of Medical Sciences, Ilam, Iran; 3 Department of Clinical Epidemiology, Ilam University of Medical Sciences, Ilam, Iran

**Keywords:** Suicide, Comorbidity, Suicidal ideation, Mental disorders

## Abstract

**OBJECTIVES:**

Little information exists on the association between comorbidities of mental disorders and suicidal ideation in developing countries. The current study examined the relationship between the presence of comorbid mental disorders and suicidal ideation in the adult population.

**METHODS:**

This cross-sectional study was conducted using the cluster random sampling method in 3 steps. Data were collected from a household assets survey and the self-administered 28-item General Health Questionnaire as first step in screening, and the Persian version of the Diagnostic and Statistical Manual of Mental Disorders, Fourth Edition - Text Revision was used in the second stage to determine the prevalence of mental disorders. Bivariate and multivariate analysis were used to investigate the associations between mental disorders and suicidal ideation.

**RESULTS:**

Of the 763 participants, 199 (26.1%) had 1 or more mental disorder. Forty-two (71.4%) subjects with comorbidities had a history of suicidal ideation, whereas 59 (7.7%) of all participants had a history of suicidal ideation. We found that major depressive disorder and obsessive-compulsive disorder were the most predictive of suicidal ideation in both sexes. The odds ratio for suicidal ideation associated with having 3 comorbid disorders was 2.70 (95% confidence interval [CI], 1.40 to 14.12) in males and 3.06 (95% CI, 1.25 to 15.22) in females.

**CONCLUSIONS:**

Consistent with pervious data, our results confirmed that mental disorders and comorbidities of mental disorders were important predictors of suicidal ideation. Our findings are very useful for applied intervention programs to reduce the suicide rate in regions in which it is high.

## INTRODUCTION

According to the World Health Organization, mental and psychological well-being is a key element for achieving good mental health [1,2]. The health care system can improve the mental health of the people through the promotion of mental well-being and by providing care to people affected by mental disorders [3]. Mental disorders have been reported in 90% of deaths from suicide, and an estimated 2-15% of deaths from suicide have been linked to major depression [4]. Moreover, the risk of suicide is higher in people who suffer from depression and at least one other psychiatric disorder, such as an anxiety disorder or bipolar disorder, at the same time [5]. Therefore, to elucidate the association between suicidal behavior and mental disorders, it would be very helpful to clarify the effects of comorbid mental disorders. Some studies have evaluated the relationship between mental disorders and suicide attempts [4,6] but few studies have evaluated the degree to which comorbid mental disorders are predictive of suicidal ideation.

In a previous study of mental disorders in Iran, Noorbala et al. [7] in 2004, in a study with 35,014 participants in which the 28-item General Health Questionnaire (GHQ-28) was used, reported that 20.1% of Iranians had a mental disorder. Moreover, Mohammadi et al. [8] in 2005 and Rahimi-Movaghar [9] in 2014 reported prevalence rates of 17.1 and 23.6%, respectively, in the population over 15 years of age. The prevalence of mental disorders in China and Nigeria was reported to be 12.0 and 13.2%, respectively, which is lower than the figures reported for Iran [10,11]. Factors such as female and living in urban sittings have been found to be predictors of risk for mental disorders in Iran in previous studies [12-14].

Promoting mental health in Iran requires recognizing the current health status of the country through survey studies, including the present study, as it was conducted in Ilam Province, which is the highest ranked in terms of suicide death among the 31 provinces in Iran [15,16]. In the current study, we aimed to determine the rate of mental disorders by age and sex and to identify the presence of comorbid psychiatric disorders according to the Diagnostic and Statistical Manual of Mental Disorders, Fourth Edition - Text Revision (DSM-IVTR) to boost our knowledge about ways that we can intervene in such situations. Moreover, we aimed to examine the association of suicidal ideation with comorbid DSM-IVTR mental disorders in the adult population.

## MATERIALS AND METHODS

### Study design

In this cross-sectional study, from June 2016 to March 2017, we enrolled people aged 15 years and over from all 10 cities in Ilam Province. The clustered random sampling method in 3 steps was used to enroll the subjects.

### Sample

The population of Ilam Province in 2016 according to the statistics provided by the health system was 623,235, of which 80% lived in 10 urban areas. Using the initial estimates, 763 residents were selected. In the initial step, we stratified the sample based on 10 cities according to the population in each city; according to this, the number of clusters (households) in each city was determined (Eyvan: 34; Ilam: 191; Chardavol: 11; Sirvan: 11; Malekshahi: 15; Mehran: 18; Badreh: 7; Abdanan: 30; Dehloran: 42; Darrehshahr: 20), so that each cluster was represented by 2 subjects. After this, the geographical area in each city based on the number of clusters was determined, with each geographical area comprising 5 clusters. We selected samples in clusters and geographical areas in all cities using the random sampling method.

### Diagnostic assessment and instruments

#### The 28-item General Health Questionnaire

Data collection was conducted using a household assets survey and the self-administered GHQ-28, which was used as the first step in screening participants for mental disorders. In 1979, Goldberg and Hillier developed this questionnaire to screen for severe depression, somatic symptoms of distress, social dysfunction, and anxiety and insomnia. The GHQ-28 has been translated into 38 different languages worldwide, and the validity and reliability of the GHQ-28 questionnaire have been demonstrated in many different countries. The validity and reliability of the Persian version of the GHQ-28 tools were confirmed in Iran by Noorbala & Mohammad [17] in 2009. In that study, the sensitivity and specificity of a GHQ-28 cut-off score for subscales 6 was 84.7 and 93.8%, respectively. In this study, a cut-off point of 23 was used to detect people with mental disorders. In each of the 4 subcategories of the GHQ-28, a cut-off score of 6 was considered indicative of a mental disorder. In-person interviewing was used to further investigate all participants with a score above the cut-off.

### Structured clinical interview for Diagnostic and Statistical Manual of Mental Disorders, Fourth Edition - Text Revision

After the screening stage, all contributors who had a score of 23 or above on the GHQ-28 were contacted and the Persian version of the DSM-IVTR was applied in the second stage. In this stage, the prevalence of psychiatric disorders, epilepsy, and mental retardation was assessed in participants referred from the first stage. Additionally, participants were assessed about suicidal ideation, which was defined as having thought about committing suicide. The Persian translation of the DSM-IVTR has shown acceptable to good reliability and validity indices [18]. Quality control was applied before and after completion of the questionnaires, and the comprehensibility and duration of the interviews were also checked. All completed interviews were checked to ensure that none corresponded to subjects with incomplete or unclear questionnaires. The clinical interviews were performed by 25 trained clinical psychologists over the course of 10 months.

### Statistical analysis

The data were analyzed using SPSS version 20.0 (IBM Corp., Armonk, NY, USA). We used cross-tabulations to estimate the prevalence of mental disorders and suicidal ideation among respondents. In the next step, we stratified outcomes by sex, and the chi-square test was used for risk estimation in models in which we recoded the variables to be dummy predictor variables for each group based on the number of comorbid disorders. Odds ratios (ORs) and confidence intervals (Cls) for the ORs were estimated using bivariate and multivariate analysis. In all models, marital status, educational level, occupation, and age group were included as covariates.

## RESULTS

### General characteristics of persons suspected to have a psychiatric disorder and suicidal ideation

A total of 763 persons aged 15 years and above were screened; the mean age of respondents was 37.90±14.20, ranging from 15-88 years. The participants were predominantly female (423, 55.4%), in the 26-45 age group (373, 48.9%), married (579, 75.9%), unemployed (461, 60.4%), and with an educational level higher than a diploma (253, 33.2%). All participants took the GHQ-28 as an evaluation. Our results showed that the prevalence of psychiatric disorders in females, married subjects, and unemployed persons was higher than among their counterparts. The pooled rate of suicidal ideation was 7.7%, with a higher rate observed in females, the 26-45 age group, married persons, and unemployed persons (Table 1).

### Sex- and age-specific rates for mental disorders

The prevalence rates of DSM-IVTR mental disorders by sex, age group (15-25, 26-45, 46-65, 65+) are shown in Table 2. In males, the most common disorders observed were anxiety disorder (18.2%), followed by major depressive disorder (MDD) (17.4%), obsessive-compulsive disorder (10.0%), phobia disorder (5.0%), and somatization disorder (4.4%). A similar pattern was observed in females, with the highest prevalence observed for anxiety disorder (23.6%), followed by MDD (22.7%), compulsive disorder (13.9%), phobia disorder (10.4%), and psychotic disorder (6.1%).

The prevalence of MDD in females increased uniformly with age, with a nearly two-fold increase from the 46-65 to the 65+ age group. The sex-specific rates for mental disorders maintained the same general patterns that were observed for the overall prevalence rates.

### Associations of mental disorders with suicidal ideation

We used bivariate analysis to investigate the associations of comorbid mental disorders and suicidal ideation. We found that MDD and compulsive disorder were the most predictive of suicidal ideation in both sexes, but a stronger association was observed in females. We controlled for possible confounding effects (marital status, educational level, occupation, and age group) of DSM-IVTR mental disorders with multivariate analysis. The effects of all DSM-IVTR mental disorders were lower than in the corresponding bivariate analysis for all categories. According to the multivariate analysis, MDD was the strongest predictor of suicidal ideation in both sexes and increased the odds of suicidal ideation by about 7 and 8 times in males and females, respectively (Table 3).

### Associations of comorbid mental disorders and suicidal ideation

Among the 763 participants, 199 (26.1%) had 1 or more mental disorder. We compared suicidal ideations in all subjects and samples according to the presence of comorbid disorders. The results showed that 42 (71.4%) subjects with comorbid mental disorders had a history of suicidal ideation, while 59 (7.7%) of all participants had a history of past suicidal ideation, and the prevalence of suicidal ideation in subjects with no mental disorder was 1.8% (n=10). Moreover, a history of any mental disorder (1 or more) was even more common among respondents with suicidal ideation (88.6%). Table 4 presents a comparison of the history of suicidal ideation according to different degrees of mental disorder comorbidity in both sexes. The ORs for having 2 comorbid disorders in males and females were 1.56 (95% CI, 1.02 to 23.01) and 1.80 (95% CI, 1.02 to 26.12), respectively. For 3 comorbid disorders, the ORs were 2.70 (95% CI, 1.40 to 14.12) in males and 3.06 (95% CI, 1.25 to 15.22) in females. Our results showed that the presence of 2 or more comorbid psychiatric disorders increased the risk of suicidal ideation in both sexes (Table 4).

## DISCUSSION

We conducted this study to estimate the prevalence and comorbidities of DSM-IVTR mental disorders and their associations with suicidal ideation in the adult population. Our results showed that 23% of participants with comorbid psychiatric disorders had ever experienced suicidal ideation, in contrast to 1.8% of all participants. The prevalence of 2 or more comorbidities in all participants was 22.2, and 71.4% of those who reported suicidal ideations were found to have comorbid DSM-IVTR mental disorders. This is in agreement with previous studies showing that previous suicide attempts and suicidal ideation are more prevalent in individuals with psychiatric disorders. Psychological autopsy studies have suggested that in suicide interventions in both developing countries and those with low socioeconomic status, a key risk factor is the presence of psychiatric disorders [19,20].

In this study, bivariate analysis was used to predict suicidal ideation in those with DSM-IVTR mental disorders. Although an association with suicidal ideation was observed for all 11 disorders, MDD and compulsive disorder showed the strongest associations with suicidal ideation, and increasing the odds of suicidal ideation by about 6 times. The lowest ORs were observed in manic disorder, panic disorder, and mental retardation. Our results are consistent with those of Nock et al. [21] and Cavanagh et al. [22] found that 80% of those who attempted suicide had a prior mental disorder. We controlled for possible confounding effects of DSM-IVTR mental disorders through a multivariate analysis. All ORs were lower, but MDD remained the strongest predictor, increasing the odds of suicidal ideation by about 7 times. In the study of Nock & Kazdin [23], the strongest predictor for suicide was depression.

In the present study, we found that the presence of comorbid psychiatric disorders was significantly associated with the risk of suicidal ideation. Other studies have shown that suicidal ideation and attempted suicide were more prevalent in adults with more than one mental disorder [24,25]. Two explanations have been proposed for this phenomenon; first, disorders such as compulsive disorder and psychotic disorder that are correlated with suicidal ideation are comorbid with MDD [21,26]. The second interpretation for this pattern is that people who suffer from multiple sources of distress may not be able to escape from them, eventually resulting in suicidal ideation and suicide attempts.

This study had important limitations. First, the diagnostic instrument was the summary form of the DSM-IVTR, which did not include all mental disorders. Second, we did not investigate suicide attempts in participants and their association with mental disorder comorbidities.

In summary, previous studies of the associations between suicide and comorbidities of mental disorders are scarce, especially in Iran. The present study offers useful results about the relationship between DSM-IVTR mental disorders and suicidal ideation in Ilam Province, Iran. Our findings will prove useful for developing appropriate preventive interventions against suicide in Ilam Province, where the population is more at risk for suicide than in other provinces in Iran.

## Figures and Tables

**Table 1.** Demographic characteristics of persons suspected to have a psychiatric disorder and those with suicidal ideation

Variable	Total	Suspected based on GHQ-28	Suicidal ideation
Sex			
Male	340 (44.6)	71 (20.9)	26 (3.4)
Female	423 (55.4)	126(29.8)	33 (4.3)
Age (yr)			
15-25	143 (18.7)	33 (23.1)	3 (0.4)
26-45	373 (48.9)	100(26.8)	39 (5.1)
46-65	216 (28.3)	55 (25.5)	13 (1.7)
65+	31 (4.1)	9 (29.0)	4 (0.5)
Marital status			
Married	579 (75.9)	153 (26.4)	47 (6.2)
Single	184 (24.1)	44 (23.9)	12 (1.6)
Educational level			
Illiterate	84 (11.0)	23 (27.4)	6 (0.8)
Elementary	95 (12.5)	33 (34.7)	10(1.3)
Secondary	110 (14.4)	36 (32.7)	9(1.2)
Diploma	221 (29.0)	57 (25.8)	20 (2.6)
Above diploma	253 (33.2)	48(19.0)	14(1.8)
Occupation			
Employed	302 (39.6)	53 (17.5)	24 (3.1)
Unemployed^1^	461 (60.4)	144 (31.2)	35 (4.6)
Total	763 (100)	197(25.8)	59 (7.7)

Values are presented as number (%). GHQ-28, 28-item General Health Questionnaire.

1 Persons with no official job.

**Table 2.** Sex- and age-specific rates for DSM-IVTR mental disorders in both sexes

DSM-IVTR disorders	Age (yr)								Prevalence (%)	
	15-25		26-45		46-65		65 +			
	Male (n=60)	Female (n=83)	Male (n=159)	Female (n=214)	Male (n=105)	Female (n=111)	Male (n=16)	Female (n=15)	Male (n=340)	Female (n=423)
Major depressive	8 (13.3)	12 (14.5)	28 (17.6)	49 (22.9)	20 (19.0)	29 (26.1)	19 (5.4)	6 (40.0)	17.4	22.7
Manic	3 (5.0)	6 (7.2)	5 (3.1)	10 (4.7)	1 (1.0)	4 (3.6)	0 (0.0)	0 (0.0)	2.6	4.7
Anxiety	7 (11.7)	14 (16.9)	33 (20.8)	46 (21.5)	18 (17.1)	33 (29.7)	4 (25.0)	7 (46.7)	18.2	23.6
Panic	0 (0.0)	6 (7.2)	6 (3.8)	18 (8.4)	4 (3.8)	11 (9.9)	1 (6.2)	4 (26.7)	3.2	9.2
Compulsive	9 (15.0)	10 (12.0)	16 (10.1)	26 (12.1)	7 (6.7)	22 (19.8)	2 (12.5)	1 (6.7)	10.0	13.9
Phobia	3 (5.0)	9 (10.8)	10 (6.3)	18 (8.4)	3 (2.9)	15 (13.5)	1 (6.2)	2 (13.3)	5.0	10.4
Psychotic	0 (0.0)	1 (1.2)	5 (3.1)	13 (6.1)	1 (1.0)	7 (6.3)	2 (12.5)	5 (33.3)	2.4	6.1
Epilepsy	0 (0.0)	0 (0.0)	1 (0.6)	1 (0.5)	1 (1.0)	3 (2.7)	2 (12.5)	1 (6.7)	1.2	1.2
Symptoms of organic brain	2 (3.3)	1 (1.2)	5 (3.1)	5 (2.3)	1 (1.0)	4 (3.6)	2 (12.5)	4 (26.7)	2.9	3.3
Mental retardation	0 (0.0)	1 (1.2)	4 (2.5)	2 (0.9)	0 (0.0)	3 (2.7)	1 (6.2)	0 (0.0)	1.5	1.4
Somatization	3 (5.0)	1 (1.2)	7 (4.4)	9 (4.2)	3 (2.9)	3 (2.7)	2 (12.5)	2 (13.3)	4.4	3.5

Values are presented as number (%). DSM-IVTR, Diagnostic and Statistical Manual of Mental Disorders, Fourth Edition - Text Revision.

**Table 3.** Bivariate and multivariate associations between suicidal ideation and DSM-IVTR mental disorders by sex

DSM-IVTR disorders	Males				Females			
	Bivariate models		Multivariable models^1^		Bivariate models		Multivariable models^1^	
	OR (95% CI)	X^2^-test	OR (95% CI)	X^2^-test	OR (95% CI)	X^2^-test	OR (95% CI)	X^2^-test
Major depressive	8.25 (1.03,15.90)	5.82^*^	7.02 (1.02,16.25)	5.53^*^	10.21 (1.63,17.05)	6.25^*^	8.23 (1.28,16.03)	5.96^*^
Mania	0.35 (0.25, 2.31)	1.88	0.33 (0.02, 2.57)	1.91	0.32 (0.03, 2.28)	2.01	0.31 (0.02, 2.36)	1.85
Anxiety	1.88 (0.52,6.10)	1.02	1.55 (0.48, 6.60)	1.03	1.92 (0.58, 6.90)	0.58	1.60 (0.45, 6.63)	0.68
Panic	0.78 (0.25, 2.85)	0.58	0.62 (0.32, 3.01)	0.45	0.85 (0.36, 2.90)	0.55	0.62 (0.31, 3.22)	0.42
Compulsive	6.02 (2.33,18.60)	10.40^*^	5.13 (2.02,16.25)	8.10^*^	7.48 (2.03,18.12)	12.30^*^	6.02 (2.08, 20.0)	11.90^*^
Phobia	1.15 (0.43, 2.98)	0.07	1.15 (0.41, 4.22)	0.06	1.18 (0.46,3.12)	0.07	1.12 (0.28, 2.98)	0.05
Psychotic	1.84 (0.58,5.11)	1.02	1.62 (0.58, 5.31)	0.9	2.28 (0.88, 8.13)	1.25	1.69 (0.68, 5.22)	1.12
Epilepsy	2.70 (0.48,13.95)	1.62	2.23 (0.28,16.02)	1.08	3.12 (0.88,15.0)	1.66	2.66 (0.92,17.15)	1.4
Symptoms of organic brain	2.14 (0.85, 7.35)	1.41	2.02 (0.85, 7.13)	1.6	2.90 (0.96,8.12)	1.92	2.30 (0.82, 7.22)	1.6
Mental retardation	0.80 (0.28,8.12)	0.08	0.78 (0.39, 7.25)	0.08	0.88 (0.08, 9.98)	0.09	0.82 (0.07, 8.25)	0.08
Somatization	2.85 (1.00, 9.22)	4.66^*^	2.13 (1.01,10.15)	4.80^*^	3.90 (1.03,12.02)	4.90^*^	3.55 (1.02,10.01)	4.85^*^

DSM-IVTR, Diagnostic and Statistical Manual of Mental Disorders, Fourth Edition - Text Revision; OR, odds ratio; CI, confidence interval.

1 In the models, suicidal ideation is the dependent variable and all models controlled for marital status, educational level, occupation, and age group.

* p=0.05 by 2-sided test.

**Table 4.** Association between suicidal ideations and comorbidities of DSM-IVTR mental disorders by sex

DSM-IVTR disorders (n)	Suicidal ideation			
	Males		Females	
	OR (95% CI)	X^2^-test	OR (95% CI)	X^2^-test
1	Reference		Reference	
2	1.56 (1.02,23.01)	7.55^***^	1.80 (1.02, 26.12)	8.28^***^
3	2.70 (1.40,14.12)	8.27^***^	3.06 (1.25,15.22)	11.77^***^
≥ 4	5.11 (2.85,11.02)	11.90^***^	5.98 (2.68, 21.58)	11.66^***^
Total comorbidities (2 or more)	4.62 (2.60,7.12)	10.01^***^	5.90 (3.01,26.12)	10.80^***^

DSM-IVTR, Diagnostic and Statistical Manual of Mental Disorders, Fourth Edition - Text Revision; OR, odds ratio; CI, confidence interval.

*** p=0.001 level by 2-sided test.
